# Trace fossils as mechanical discontinuities in shales, insight for the generation of bedding-parallel veins (BPV)

**DOI:** 10.1038/s41598-024-63665-w

**Published:** 2024-06-03

**Authors:** Alain Zanella, Remigio Ruiz, Augusto N. Varela, Mariano G. Arregui

**Affiliations:** 1 Laboratoire de Planétologie et Géosciences, LPG-UMR 6112 CNRS, Le Mans Université, Université d’Angers, Nantes Université, Avenue Olivier Messiaen, 72085 Le Mans, France; 2 YPF-Tecnología S.A. (Y-TEC), National Research Council (CONICET), Avenida del Petróleo Argentino s/n, 1923 Berisso, Argentina

**Keywords:** Bedding-parallel veins, Ichnological features, Fluid migrations, Natural fracturing, Sedimentary facies, Structural geology, Sedimentology, Palaeontology

## Abstract

Understanding shale petrophysical parameters is of interest due to its direct implications as cap rocks for CO_2_ or hydrogen storage, waste depositions, and as unconventional reservoirs. The generation and propagation of natural and induced fracture networks in such rocks is highly dependent on the mechanical behavior linked to several sedimentological parameters, as lithological discontinuities or bioturbation. This study is focused on a different sedimentological parameter that consists of trace fossils and their implication on the generation of fluid-assisted fractures, called bedding-parallel veins. In the Austral-Magallanes Basin, Southern Patagonia, Argentina, both geological features, *Skolithos* Ichnofacies (doomed pioneers trace fossils) and bedding-parallel veins, are numerous, especially at the top of the turbiditic bodies. The trace fossils exhibit U-shaped vertically oriented burrows composed of clean sandstone, partially cemented by calcite, and a *spreite* in the central part with heterogenous laminated siltstone. Bedding-parallel veins are composed of calcite fibers with some pyrite grains and bitumen. They are located on the top of the trace fossils along the lithological discontinuity between the turbiditic bodies and the impermeable shales. On their surfaces, a radial pattern starts growing from the trace fossils. Moreover, the number of bedding-parallel veins is dependent on the bioturbation intensity. With this study, we infer that trace fossils represent ichnological mechanical discontinuities (IMD) that have a key role in the generation and development of bedding-parallel veins. By correlation, we also suggest that these geological features must be thoroughly studied, especially regarding their potential for the development of induced fracturing networks.

## Introduction

Bedding-parallel fibrous veins (BPV) are very common worldwide in sedimentary basins on Earth, especially within or near shales^1–3^ as well as in lacustrine rocks on Mars^4^. The origins of BVP generation is still debated. While there is consensus about the involved parameters, such as the fluid overpressure buildup^5^, the maturation of organic matter and fossils^6,7^, the force of crystallization^8,9^, and the tectonic stresses^3,10^, there is no evidence in favor of one parameter being more important than the others. On the other hand, it is well known that sedimentological parameters, such as bioturbation, change the primary petrophysical characteristics (i.e. porosity and permeability) of the sediments, improving or degrading their reservoir quality^11–17^. The modification of the petrophysical characteristics by trace fossils also controls the migration of fluids (water, oil, natural gas, CO_2_ and nitrogen) into the reservoirs^11,13,18^. Although the trace fossil impact on BPVs is a new idea, it has been well established that fossils in general may be initiation points of BPVs^6^.

The comprehension of the BPV formation is of great importance because of their role in the fluid migration pathways or barriers within very low permeable rocks, such as shales. In view of the potential of shales in reservoir exploitation for hydrocarbons as well as their role as reservoirs or cap rocks for CO_2_ or hydrogen storage, it is extremely relevant to study these geological features beforehand.

The aim of this work is to demonstrate the importance of ichnological mechanical discontinuities (trace fossils) on fluid migrations as well as on BPV generation and distribution in shales successions based on detailed analysis on “world-class” outcrops.

## Geological setting

**Figure 1:**
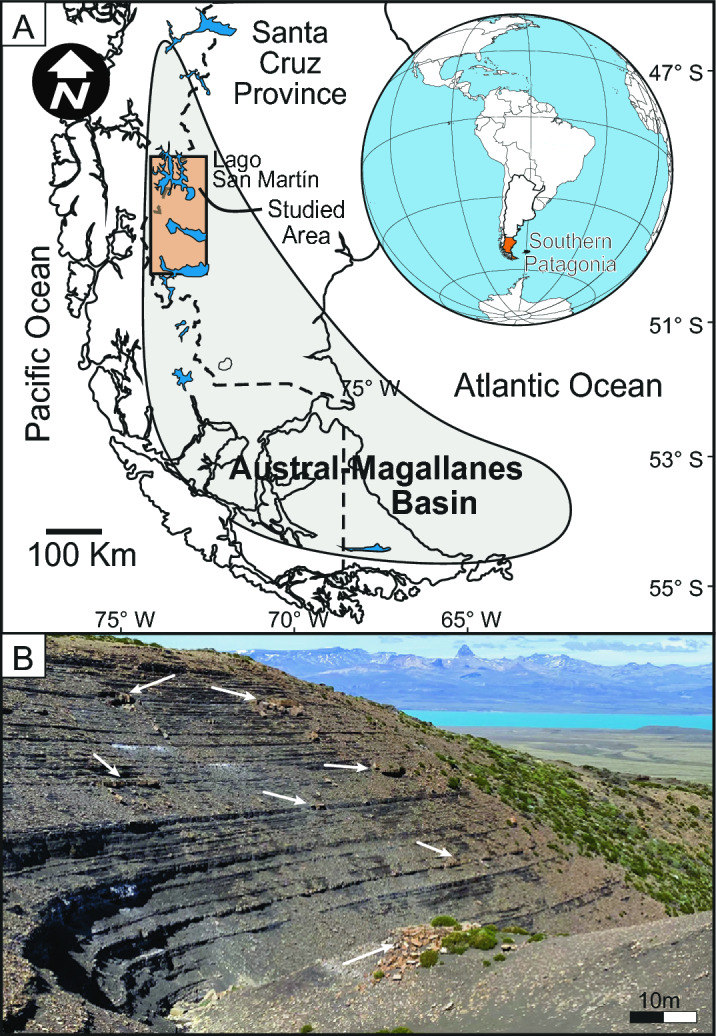
(**A**) Map of the study area (designed with CorelDRAW 2017 v.19.1.0.419). (**B**) Outcrop view shales of the Río Mayer Fm., white arrows show the main turbiditic bodies.

The Austral-Magallanes Basin, Southern Patagonia Argentina (Fig. 1A), is a multiphase basin that began with a rift stage (uppermost Jurassic-early Cretaceous), followed by a sag stage (early Cretaceous- mid-Cretaceous), and finally a foreland stage since mid-Cretaceous with a NNE-SSW trend^19,20^. Black shales of the Río Mayer Fm (Berriasian-Albian), which are the main source rocks of the basin, were deposited during the sag stage, reaching up to 1000 m thick (Fig. 1B)^21–23^. The lower section is dominated by laminated black shales interbedded with marl levels accumulated in an outer shelf setting. The middle section is composed of intensely bioturbated dark marls and shales. The upper section is constituted by massive and laminated black shales intercalated with very fine- to fine-grained sandstones, interpreted as an outer shelf with distal low-density turbidity current deposits, related to the distal deltaic influence^21^. The most common trace fossils in outer shelf environments in the Palermo Aike Formation are *Zoophycos*. isp., *Bergaueria* isp., *Phycosiphon* isp., *Chondrites* isp., *Paleophycus* isp., and allochthonous *Teredolites* isp.^22,24^ These trace fossils are usually present at levels displaying low ichnodiversity and low bioturbation intensity, interpreted as belonging to the *Zoophycos* ichnofacies, typical of low energy and dysoxic conditions^22,24^. The presence of trace fossils interpreted as doomed pioneers in Rio Mayer/Palermo Aike Formation was reported from outcrops in Santa Cruz province by^22^, and in the subsurface in Tierra del Fuego province by^24^. In this work, we report the presence of a low diversity trace fossil assemblage composed by *Diplocraterion* isp. and *Arenicolites* isp. in the top of turbiditic levels. In the Lago San Martín area (Fig. 1), the presence of BPV in the form of "calcite beef" was reported in the Río Mayer Formation and was attributed to fluid overpressure during hydrocarbon generation or migration^25,26^.

## Trace fossils and BPV relationship

The outcrops of the Río Mayer Formation in the studied area (Ea. La Federica, Fig. 1A) show a succession dominated (more than 80%) by black to dark gray laminated claystones and mudstones (facies Fl; Fig. 1B) interbedded by isolated lenticular bodies of whitish massive very fine- to medium-grained sandstones (Sm) and gray siltstone- to very fine-grained marlstone (Lm; Fig. 1B). These silty facies acquire a brown color externally due to weathering (i.e. oxidation). Frequently, some lenses show diffuse current ripple towards the tops (Sr and Lr facies). This succession has been interpreted as a product of sedimentation in an external shelf environment (Fl facies) influenced by distal turbiditic flows of the deltaic front (Sm and Lm facies) in agreement with previous outcrops^21–23^ and subsurface studies^24^.

**Figure 2:**
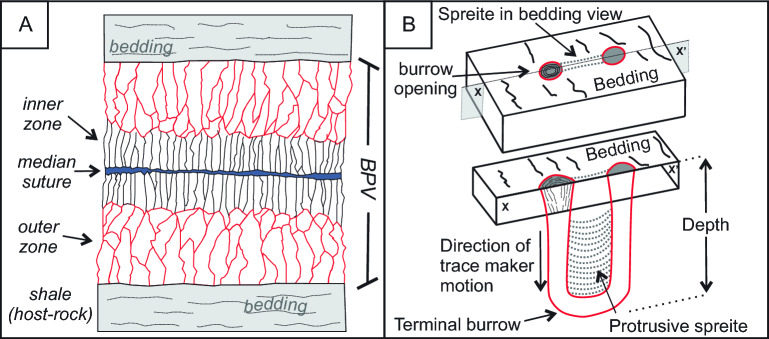
(**A**) Detailed model of a Bed Parallel Vain (BPV) structure. (**B**) Detailed model of a U-Tube trace fossil with *spreite* (*Diplocraterion*), modified from^27^^.^

BVP are distributed through the entire succession of the Rio Mayer Fm, have a thickness range from a few millimeters to a few centimeters thick, and have a typical length of a decimeter (Fig. 2A). The BPV composition is made up of calcite fibers with bitumen, some pyrite grains, and shale fragments highlighted by the cone-in-cone typical internal structure, clearly visible in most BPV (Supplementary Fig. S1). The BPV distribution is not random, and they are mainly localized at: (i) facies boundaries, (ii) at stratigraphic nodule levels; and (iii) in turbiditic siltstone to very fine-grained sandstone bodies, especially on the upper boundary.

**Figure 3:**
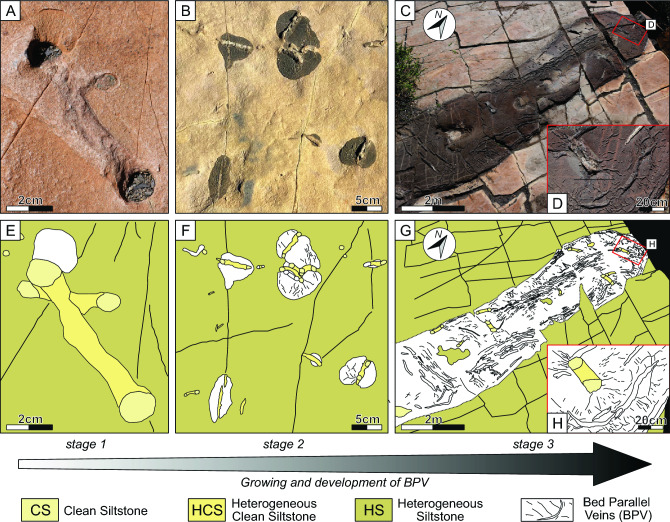
(**A**) A detailed plan view picture of two *Diplocraterion* trace fossils. (**B**) Detail plant view picture of abundant trace fossils with isolated BPV associated. (**C**) Planar view picture of tongue-shaped BPV with associated trace fossils showing lineal and radial fluid patterns. (**D**) Detailed zoom view (red box) of (**C**) showing *Diplocraterion* trace fossils and the radial pattern of fluid injection. (**E–H**) Sketch of this figure (**A–D**) showing the different stages of BPV growth and development and fluid pattern migration. *BI* bioturbation index.

**Figure 4:**
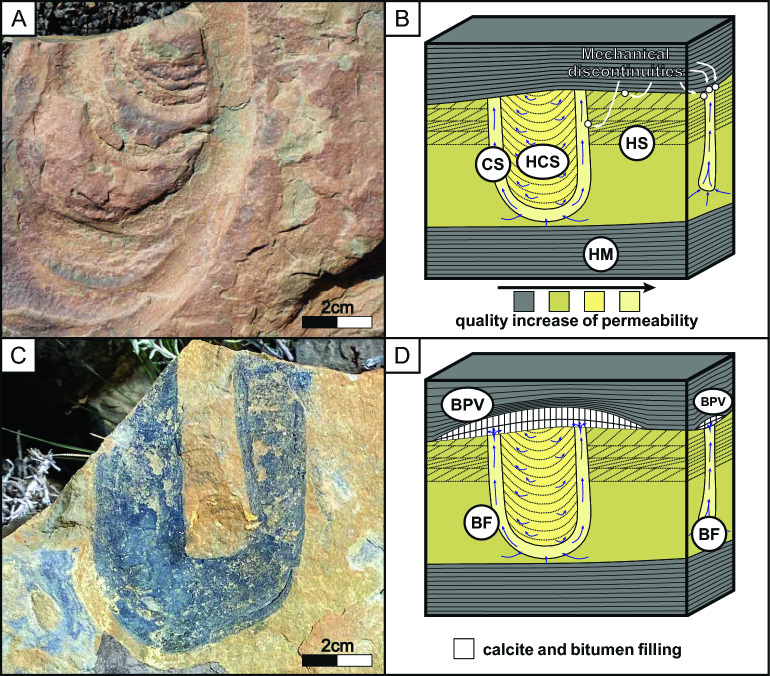
(**A**) *Diplocraterion* vertical view showing the tube burrows and *spreite*. (**B**) Sketch of a turbiditic body in the shale with *Diplocraterion* trace fossils, where *HM* heterogeneous mudstone, *HS* heterogeneous siltstone, *CS* clean siltstone, *HCS* heterogeneous clean siltstone, showing the different flow pathways (blue arrows) inside the trace fossils and in the wall of the trace fossils (lateral view). (**C**) U-shaped trace fossil vertical view showing black colored tube burrows and the *spreite* cemented with calcite and bitumen. (**D**) Sketch of a turbiditic body in the shale with *Diplocraterion* trace fossils, after the development and growth of the BPV, showing BPV and the trace fossils filled (TFF) by calcite and bitumen.

Trace fossils are recognizable within and on the top of turbiditic bodies. The most abundant trace fossils are U-shaped, vertically oriented burrows, that appear as paired circular openings on the bedding surface. These burrows can present lamination on the inner part of the U shape, called *spreite* (*Diplocraterion* isp.) or without lamination (*Arenicolites* isp.) in vertical planes (Figs. 2B, 3A–D, 4A, C). The closely spaced lamination of the protrusive *spreite* is the result of the successive vertical shift of the burrows, that is usually attributed to how the producer reacts to changes in sedimentation rates^27,28^ (Fig. 2B).

The trace fossils described have a 3–6 cm width and penetrate into the substrate 10 to 15 cm. The burrow diameters are between 1 and 1.5 cm, and the walls are smooth. The tubes of these U-shaped trace fossils are filled with host clean sediment without lamination (clean homogeneous siltstones, Fig. 4A, B); whereas the *spreite*, is filled with a clean heterogeneous siltstone (Fig. 4A, B). Often, the U-shape tubes and the external part of the *spreite* are cemented by calcite and bitumen (Fig. 4C). The producers of *Arenicolites* and *Diplocraterion* are usually interpreted as suspension feeders^27,28^. The distribution and abundance of trace fossils on the top of the turbiditic deposits vary from place to place, reaching up to 30 trace fossils per square meter, with a bioturbation index (BI) that ranges from 1 to 3^29^ (Fig. 4A–D). These trace fossils are grouped in *Skolithos* Ichnofacies; which are typically from energetic marginal marine environments or could be represented as doomed pioneers in turbiditic current deposits^22,24,30^.

At the top of the turbiditic bodies, BPV are systematically related to the upper sections of the trace fossils (Fig. 4A, B). Not all trace fossils exhibit this relationship, but from a top view, the relationship between the localization of BPV and trace fossils is clearly visible (Fig. 4B). Moreover, as described above, the trace fossil cementation is mainly of the same composition as BPV with a calcite filling, and in some cases, bitumen is present as well (Fig. 4C). The main relationships found are: (i) cementation observed on the trace fossil vertical views has the same composition as BPV (calcite and bitumen; Fig. 3C); (ii) the initial points from which BPV appears to grow are trace fossils (Fig. 4A, B) and (iii) once the growing and development of the BPV continues, their extent is strongly controlled by the presence of trace fossils (Fig. 4C, D).

## Implications of trace fossils in the BPV developments

### Lithological and ichnological mechanical discontinuities

Lithological mechanical discontinuities (LMD) in shale successions are numerous, and they have a great impact on the rock´s mechanical behavior. The presence of LMD is of great importance for applications in earth sciences, both in terms of the mechanical and hydraulic properties of individual discontinuities and fractured rock masses^12,13^. However, despite LMD being in the spotlight, there are no mentions in the literature regarding the effect of trace fossils on mechanical discontinuities and their mechanical and hydraulic properties.

Although the effect of body fossil as mechanical discontinuities has been recognized in the literature^6^, the general assumption is that the contrast of lithology makes them act mainly as LMD. On the other hand, the petrophysical properties of the infill of the trace fossils and the disposition of the wall of the burrows makes them ideal pathways and injection points for fluid migration. All these reasons are sufficient to propose a new category of ichnological mechanical discontinuities (IMD).

As was recognized in the detailed description and mapping of BPV described below, there is a cause-and-effect relationship between mechanical discontinuities, both LMD and IMD, and BPV distribution and development. IMD work as vertical mechanical discontinuities that efficiently collect fluids and then inject them into LMD. In turn, LMD´s are bedding parallel mechanical discontinuities, where BPV take place and develop.

### Fluid pathway migration and BPV generation

It was recognized that fluid collection, injection, and migration pathways are one of the main factors controlling the generation and growth of BPV from the Río Mayer Fm (Figs. 3 and 4). Regarding this statement, trace fossils, as IMD, play a key role in both the collection and vertical migration through them (Figs. 3, 4), as well as in the growth and bedding-parallel connectivity of BPV (Fig. 3).

### Fluid migration through the trace fossil

Trace fossils, in this case, have better petrophysical properties such as porosity and permeability due to the passive infilling of the burrows with looser, homogeneous sediment than the host rock. Also, the activity of the producer eliminates heterogeneities in the primary sedimentary structures (i.e., CS: clean homogeneous siltstone; Fig. 4B)^15^. In turn, the vertical movements of the tracemarkers generate the *spreite* (Fig. 2B), which produces HCS: heterogeneous clean siltstone (Fig. 4B). The *spreite* works in its middle part as a vertical permeability barrier. However, the upward orientation in between the burrows generates a connection, increasing the vertical connectivity, and the fluid flow generates a bitumen filling in the outer parts of the *spreite* (Fig. 4C, D). The fluid migration pathway occurs both inside the tube and outside the *spreite* of the trace fossils (Fig. 4B), as well as at the walls of the IMD´s where there is a contrast in physical properties between the host rock and the IMD (see lateral view of Fig. 4B). Therefore, IMD works as vertical pathways for collecting and distributing fluids (Fig. 4B).

### Growth and connectivity of BPV

As indicated in the previous section, these trace fossils are vertical IMDs that control the injection of fluids until they reach bedding parallel planes (Fig. 3). It was noticed that the fluid migration pathway occurred from both the burrows and the IMD walls (Fig. 3B–F). In very extended BPV, at least two types of fluid migration patterns are recognized. On one hand, there is a BPV linear development that follows a tongue-shape with an NNE-SSW trend in accordance with the regional compressional stresses during the basin foreland stage (Fig. 3C–G)^19^. On the other hand, there is a smaller-scale local radial pattern associated with trace fossil openings, that works as injection points (Fig. 3D–H).

Thus, depending on the amount of fluid collected by the trace fossils, different stages of BPV growth and interconnections are recognized: stage (1) isolated BPV with one single injection point (trace fossil) (Fig. 3A–E); stage (2) grouped BPV with multipoint injections without interconnections (Fig. 3B–F) and stage (3) grouped interconnected BPV with multipoint injections (Fig. 3C–G). An important point to highlight is that during stages (1) and (2) the fluid migration pattern and its consequent BPV development, is governed by the local injection points generating radial patterns (Fig. 3A–E; B–F). During stage (3), in addition to these radial patterns (Fig. 3D–H), the local stress field promotes the development of the BPV with a tongue-shaped morphology whose major axis is aligned with the main stress (NNE-SSW)^19^ (Fig. 3C–G).

Trace fossils have more influence on the first BPV growth stages (e.g. stages 1 and 2, Fig. 3A, B). Consequently, the propagation of BPV along the sedimentological level is more related to the presence of a sedimentary mechanical discontinuity and thus started to be governed by other physical parameters such as tectonic stresses (Fig. 3C).

### Key physical parameters that control BPV generation

Two main groups of physical parameters govern the BPV generation: (i) parameters linked to the fluid, such as quantity, calcium content, fluid overpressure, and the crystallization force^5–7,9^ and (ii) the host rock parameters, such as organic matter content and the presence of mechanical discontinuities^1,6,10,26^. In this study, we demonstrate that the combination of both lithological and ichnological (trace fossil) mechanical discontinuities can be involved in fluid migrations through shales. Trace fossils control the 3D pattern of the fluid pathway network because of their efficiency as mechanical discontinuities. Due to fluid overpressure, these mechanical discontinuities are opened by the natural hydraulic process. Thus, this network can be “fossilized” by the mineralization of calcite if the thermodynamic conditions are conducive.

## Conclusion

In the Rio Mayer Fm., Austral-Magallanes Basin, Southern Patagonia, Argentina, bedding-parallel veins are located at mechanical discontinuities through the sedimentary succession, especially at the top of the turbiditic bodies where trace fossils are numerous. The relationship between the localization of trace fossils and bed parallel veins is clear, and we infer that trace fossils represent ichnological mechanical discontinuities that have a key role in the generation and development of bed parallel veins. Thus, due to their shape and filling, trace fossils facilitate the fluid pathway, which is able to collect and then inject the fluid at the boundaries between turbiditic bodies and shales, generating natural hydraulic fractures extending from the trace fossils. Then, this fracture is “fossilized” by the mineralization of fibrous calcite, forming a bedding-parallel vein.

Based on our observations and study, we infer that trace fossils can be considered IMD that have an important significance in (i) fluid circulation in impermeable sedimentary rocks; (ii) the localization and development of BPV and (iii) the localization and development of induced hydraulic fractures in unconventional reservoirs, as well as to the utilization of impermeable rocks for the storage of natural gases, CO_2_, and hydrogen.

### Supplementary Information

Supplementary Information.
