# Supplementary material for: Trace fossils as mechanical discontinuities in shales, insight for the generation of bedding-parallel veins (BPV)
Source: Sci Rep. 2024 Jun 3;14:12746. doi: 10.1038/s41598-024-63665-w (PMC11148038; doi:10.1038/s41598-024-63665-w)
Supplement: Supplementary file 1 — Supplementary Information. [file 41598_2024_63665_MOESM1_ESM.docx]

**SUPLEMENTARY DATA**

**Introduction**

In order to observe and describe the internal structure of the BPV's of the Austral-Magallanes Basin, we selected the BPV with tongue-shaped morphology (Fig. A), and to determine the relationship between the U-shaped fossil traces (Diplocraterion isp.) and the injection and development of the BPV, several thin sections were made at strategic locations of the structures (Fig. B). One thin section longitudinally to the U-shaped traces fossil (*Diplocraterion* isp.), which mainly covers the central spreiten and part of the burrow on the right (Figs. C and D). Another transversal one to see in detail the vertical burrow (Fig. E and F) and a last one in the parts of the BPV without fossil traces, where it grows by coalescence and migration, forming ridges (Fig. G and H).

**Methodology**

Thin sections (30 µm of thickness) were used for textural and compositional analyses using a polarization microscope Zeiss AX10 Imager.M2m from Y-TEC (YPF Tecnología). A detailed description of textural and compositional both BPV and shale host-rock components was made for each thin section. The photomosaics were made with Zeiss software in order to illustrate the thin sections completely for both parallel (PPL; Figs. C, E and G) and crossed polarized light (XPL; Figs. D, F and H).

**Thin-section descriptions**

The first two thin sections, longitudinal (Fig. B & C) and transversal (Fig. D & E) to the *Diplocaterion* isp trace fossil, show a conical shape with a fibrous calcite composition (F. Cal.) with sub-orthogonal calcite fibers compared to the bedding. Shale host rock fragments (H.R.) are abundant and show a hydraulic brecchia pattern. The abundance and relatively large size of shale host rock fragments (larger than 3 mm) is noteworthy (Fig. C and D) and the surrounding area shows abundant subsparitic blocky calcite without a clear temporal relationship (B. Cal.; Fig. C and D; Figs. E and F).

The last thin section, in the sector of the BPV that is not related to trace fossils, shows a fibrous structure of calcite (F. Cal.). The calcite fibers are arranged orthogonally to the bedding plane (Figs. G and H). Fragments of shale host rock are also identified with a maximum size of few microns. Fragments are mainly located along the planes that depict the conical structure (cone-in-cone shape) (Figs. G and H). Bitumen was also observed between fibers of calcite (Figs. G and H). Finally, local deformations of the calcite fibers are observed in the areas corresponding to the two ridges, which seem to correspond to cone-in-cone structures and part of the light brown color corresponds to bitumen that migrated during the formation of the BPV (Figs. G and H).


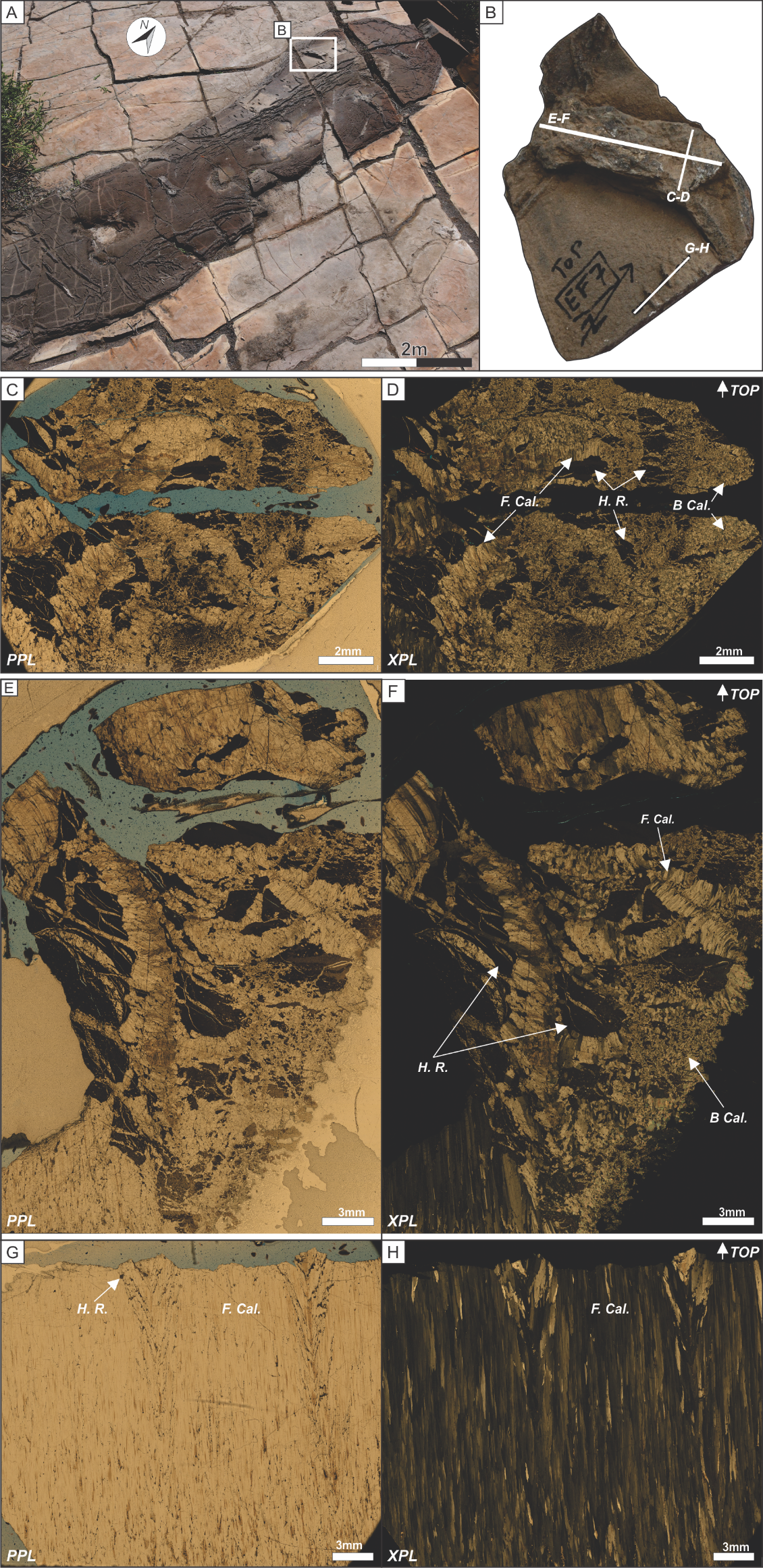


Figure A: Plan view picture of tongue-shaped BPV with associated trace fossils showing lineal and radial fluid patterns. Figure B: Location of three thin-section samples. Figure C-D: Cross-section of the BVP related to the Diplocraterion burrow (C: Natural light; D, polarized light). Figure E-F: Cross-section of the BPV related to Diplocraterion *spreite*. Figure G-H: Cross section of the BPV. PPL: Plain-Polarized Light; XPL: Cross-Polarized Light; F.Cal: Fibrous calcite; H.R: Host Rock; B.Cal: Blocky calcite.


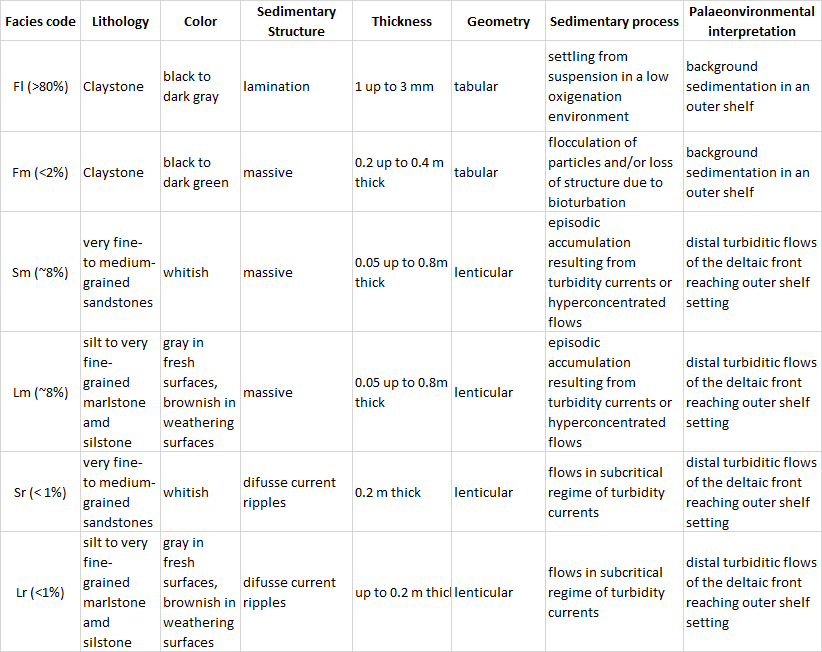
Supplementary Table 1. Sedimentary Facies Codes of Rio Mayer Formation.
